# Research on the Design of and Preference for Collection Modes of Reusable Takeaway Containers to Promote Sustainable Consumption

**DOI:** 10.3390/ijerph17134764

**Published:** 2020-07-02

**Authors:** Xiaohong Jiang, Mingyu Dong, Yuewei He, Jiayi Shen, Wenqi Jing, Nan Yang, Xiucheng Guo

**Affiliations:** 1 College of Automobile and Traffic Engineering, Nanjing Forestry University, Longpan Road 159#, Nanjing 210037, China; 15896227028@163.com (M.D.); heyueweituffy@163.com (Y.H.); sjy19990414@163.com (J.S.); jwq2450237952@163.com (W.J.); yangnanyoung@163.com (N.Y.); 2 School of Transportation, Southeast University, Si Pai Lou 2#, Nanjing 210096, China; 101002320@seu.edu.cn

**Keywords:** reusable takeaway containers, design of collection modes, preference, binary logistic regression model, sustainable consumption, food health and safety, environmental protection

## Abstract

The development of the online to offline business has accelerated the growth of the online food ordering market in China. The widespread use of disposable takeaway containers has resulted in a large amount of waste, which seriously affects the ecological environment. This paper studied the collection modes of reusable takeaway containers and the preferences of consumers and merchants. First, after two rounds of discussion and revision, four takeaway container collection modes were designed. Second, based on the survey results of consumers and merchants, a binary logistic regression model was applied to analyze the preferences of consumers and merchants. The results showed that the consumers’ delivery requirements and the current disposal of takeaway containers had a significant impact on consumers’ preferences. Consumers were more concerned about the hygienic status of the containers, food health and safety, while the merchants were more concerned about the increased costs. The promotion of collection modes requires the special consideration of the locations of dishwashing facilities and increased costs. Finally, according to the preferences and concerns of consumers and merchants, several suggestions on promoting the collection mode, such as the use of different promotion strategies for different people, the short distance of dishwashing facilities, reward systems, and food safeguard measures were proposed. This research provides guidance for decision making regarding the sustainable consumption and the promotion of reusable takeaway containers, which will contribute to resource conservation, ecological environmental improvement and sustainability.

## 1. Introduction

**Figure 1:**
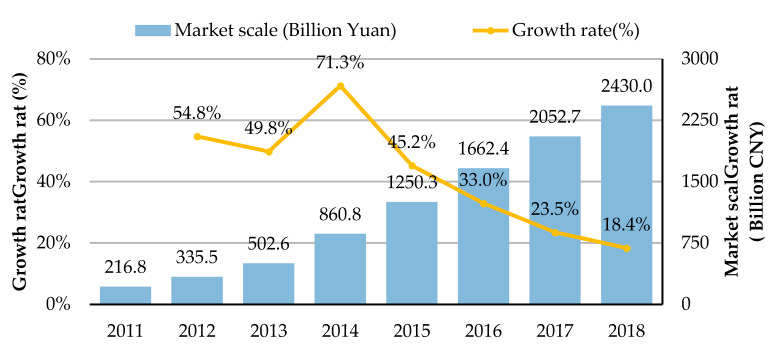
Chinese market scale of online catering takeaway from 2011 to 2018 (Data source: the analysis report on big data of Chinese catering takeaway in 2018 released by Chenzhi Technology [1]).

With the rise of the online to offline (O2O) business, the restaurant industry has undergone major changes, and the takeaway industry is growing rapidly in China. As the pace of life accelerates, work pressure is greater, and as people seek quick and convenient ways to eat, takeaway has become almost a necessity of life for the majority of office workers and students. According to an analysis of Chinese catering takeaway businesses, the takeaway market is growing [1], as shown in Figure 1. The market scale in 2018 was CNY 240 billion. The number of takeaway consumers was 256 million in 2016, 305 million in 2017 and 358 million in 2018. By June 2019, the number of takeaway consumers was 421 million, accounting for 49.3% of all Internet users. The takeaway market is not yet saturated and is still showing steady growth. More types of snacks are now on the two takeaway platforms, namely Meituan takeaway and Eleme. With the recognition of the takeaway industry, takeaway is penetrating consumer markets of all ages, such as middle-aged and elderly people who are also consuming takeaway. As takeaway deliveries skyrocket and restaurants extend their hours of operation, takeaway consumption is shifting from the midday and evening peaks [1].

With the rapid growth in the online food ordering market, such a large amount of takeaway orders has resulted in a large amount of takeaway packaging waste, which is collected in large piles and difficult to handle. The impact of this waste on the environment is a matter of concern [2,3,4,5,6,7]. According to a survey, takeaway waste accounted for 40–50% of the weight and 60–70% of the volume of the total waste from an office building every day in China [8]. There are nearly 20 million takeaway orders every day across Meituan takeaway and Eleme, which hold about 97.8% of the takeaway market share. A rough calculation shows that 240 m^3^ of takeaway garbage is generated per day [9]. In China, most of the existing takeaway packaging materials are disposable polypropylene, and a very small proportion are of paper and degradable materials [10,11,12,13,14,15,16]. Gallego-Schmid et al. evaluated the life cycle of aluminum, polypropylene and polystyrene, and concluded that polypropylene had the largest impact on the environment [17]. The problem of non-recyclable takeaway garbage has seriously affected the environment, and the garbage can be expected to continue to grow further because of the not yet saturated market. It is therefore necessary to promote sustainable development by developing and popularizing green and low-carbon takeaway containers and collection methods.

Countries are looking for ways to decrease takeaway waste, and they are focusing on the use of environmentally friendly degradable containers. Expanded polystyrene (EPS) containers have the lowest impacts due to the lower material and electricity requirements in their manufacture [18]. However, EPS is rarely recycled and mostly goes to waste incineration. As a material with a higher density and hence higher mass, but which is recyclable would have a less negative environmental impact, EPS tends to disintegrate into smaller pieces, which leads to littering. The use of such containers could effectively alleviate global warming and have less terrestrial ecological toxicity [18]. Gallego-Schmid et al. found that implementing the European Union 2025 policy on the recycling of the total number of single-use containers would reduce all the impacts by 2% (ODP, ozone depletion potential) to 60% (EP, eutrophication potential and FAETP, freshwater aquatic ecotoxicity potential), including a 33% reduction in global warming potential [18]. Biodegradable food containers are gradually emerging in the takeaway market in many countries because of their biodegradability. Many countries, such as the United States [19], Germany [20], France [21], and Japan [22] rely on policies that mandate the collection and recycling of food containers, while facilitating the abilities of individuals to separate waste. In recent years, China has been actively formulating management methods related to recycling. Shanghai has implemented the household waste management regulations from July 2019. Beijing and Nanjing will implement regulations from 2020. However, China does not yet have a law on mandatory recycling, and the difficulties with recycling still exist.

The use of reusable and non-disposable takeaway containers has become more widespread. That is, takeaway merchants provide reusable containers for consumers to use, and consumers return the containers to a collection point, thus enabling the reuse of the containers. In Japan, the reusable and non-disposable containers, such as porcelain bowls or lacquer bowls, have been implemented through two recycling modes. One mode involves consumers paying a deposit, washing the containers, and placing the containers in a fixed location where the merchants collect the containers. The second mode involves consumer washing the takeaway containers and then delivering them to a collection center. The containers are then uniformly packaged and sent to a collection center. At the collection center, the containers will be washed, disinfected and then provided for reuse [22].

Some research has been conducted on the promotion of the use of reusable takeaway containers. In an investigation of the collection of disposed containers in a Japanese university campus, it was concluded that offering a cash reward improved the collection rate of wasted containers [23]. In Japan, consumers are given subsidies to encourage such environmentally friendly practices [22]. Dorn et al. conducted an experiment examining the rental of reusable takeaway containers at select restaurants where consumers could rent reusable containers and get their rental fees back by returning the containers to a collection bin. This experiment has shown that social influence can impact the consumers’ behavior. When a small percentage of consumers rent reusable containers, others will follow suit. Another way is to add a message about renting a reusable container at the takeaway counter, but this approach has no impact on consumers’ choices [24].

At present, in the Chinese catering market, reusable containers have not been used in takeaway meals, but have been applied for dinners in the restaurant. Some scholars have studied the collection modes, which means the complete system, including the collection and processing. Yiran et al. applied for an invention patent for a collection system of reusable containers [25]. Sanwu et al. invented a collection system involving reusable containers, unmanned collection systems and mobile apps [26]. Xiaoran invented a double-layer environmental shared container and sharing system [27]. Sen et al. invented a collection cloud platform system with passive electronic labels on takeaway containers [28]. It can be seen that these designs are basically connected with merchants, consumers and delivery drivers through an information platform to process information such as the moving trajectory of the takeaway containers. Consumers return the containers to the collection bins and then the delivery drivers pick up the containers to the dishwashing center for operation.

Further research on how to promote reusable takeaway containers is needed. It is unclear whether merchants and consumers are willing to accept reusable containers and whether collection modes conflict with merchants’ interests and consumers’ need for convenience or others [24]. It is necessary to conduct further research to analyze the consumers’ and merchants’ preferences for collection modes and to identify the factors influencing their preferences. Maturos et al. conducted a performance survey and found that the ecological effect and knowledge were significant in determining young consumers’ green involvement as well as their actual purchase by using a structural equation model [29]. Babak et al. summarized five methods for researching collection behavior, namely the interview method [30,31,32], questionnaire method [33,34,35], case study method [30,36], modeling method [37,38] and the literature review method [30,33,34,39]. The interview method is mostly used in combination with questionnaires. The questionnaire method is the most common method of collecting large amounts of data. Existing research methods were used to inform this study. The choices of collection modes can be understood by using a quantitative analysis to explain the consumers’ choices from a probabilistic perspective. Discrete selection models are usually applied for this analysis. Logistic models are the most widely used. In the analysis of probabilities, the logistic model uses a fixed form that is relatively easy to explain [40]. This type of model can predict not only sampled data but also non-sampled data [41]. The logistic model is a more developed method and is widely used in psychology, sociology, economics and transportation [42,43,44,45,46,47]. For example, Zhang explored the logistics dispatcher’s preference in electric tricycle by a binary logistic model [46]. A logistic regression model will be used to explore the factors that influence the consumers’ and merchants’ preferences for collection modes in this research.

The goal of this study was to better explain what the consumers’ and merchants’ preferences for collection modes of reusable takeaway containers were and explore the factors influencing their preferences. To achieve this goal, from September 2018 to June 2019, in four cities, i.e., Nanjing, Jiangyin, Yixing and Yulin, the authors conducted a face-to-face questionnaire survey to analyze the demand, preferences, concerns and suggestions of merchants and consumers for reusable takeaway containers. Four experts were invited to provide decision support and advice on research items. We proposed effective collection modes to get more people involved. Furthermore, a logistic model was applied to analyze the consumers’ and merchants’ preferences and the influencing factors of collection modes. In combination with the concerns and suggestions, the promotion suggestions were proposed. In general, this research contributed to the promotion of the sustainable consumption of takeaway containers in China. The widespread use of disposable takeaway containers resulted in a large amount of waste, which seriously affects the ecological environment. Not only China has these problems, but maybe other countries around the world are also having similar problems. This research provides scholars in more countries with guidance for decision making regarding the sustainable consumption and the promotion of reusable takeaway containers, which will contribute to resource conservation, ecological environmental improvement and sustainability on a broader scale.

The content of the rest of this paper is organized as follows. Section 2 analyzes the advantage of the reusable takeaway containers. Section 3 states the research methodology. Section 4 describes the results of the collection modes design and regression model. Section 5 is the discussion and suggestions, and Section 6 is the conclusion.

## 2. The Advantage of Reusable Takeaway Containers

The following is an analysis of the advantages of promoting reusable takeaway containers compared to promoting the recycling of biodegradable single-use food containers.

The difficulties with recycling of biodegradable single-use food containers exist in China. First, the coated paper bowl in degradable containers has poor extrusion resistance, as soup leaks easily; thus, these containers are not suitable for Chinese foods with liquids and high temperatures. Second, the price of biodegradable containers is higher than that of non-biodegradable containers, and merchants are more inclined to choose disposable plastic containers due to their low cost. Third, the low recycling rate of biodegradable food containers is also a problem that cannot be ignored [9]. The biodegradable containers are always discarded along with household waste for incineration. Moreover, the separation of food from containers is even more costly. Even scavengers are reluctant to collect such biodegradable containers. If degradable containers cannot be easily recycled, then they do not make environmental sense either [18].

At present, in China, the reusable melamine containers have not been used in takeaway meals, but have been applied for dinners in restaurants. There are corresponding dishwashing centers to provide dishwashing and disinfection services, therefore, these centers can be used for reusable takeaway containers. From the perspective of cost and environmental protection, food containers made of low-cost straw materials and ceramics are recommended [17].

Thus, promoting reusable takeaway containers have the following advantages compared to the biodegradable single-use food containers in China. First, the reuse of reusable takeaway containers can fundamentally reduce the waste of takeaway packaging. Second, the reusable containers can hold all categories of Chinese food. Third, the dishwashing centers established in the Chinese catering market provide an infrastructure for promoting the use of reusable takeaway containers. Fourth, in the short term, the cost of reusable containers is bound to be higher than that of biodegradable containers. However, in the long term, the cost advantages of reusable meal containers will gradually become apparent based on their reusability.

## 3. Research Methodology

### 3.1. Two-Step Research Process

**Figure 2:**
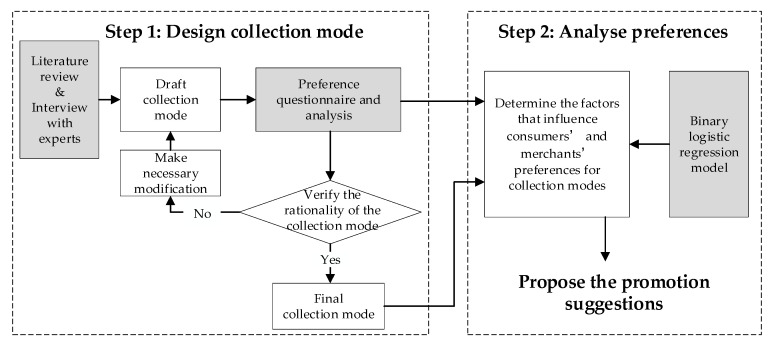
Flow chart of the research process.

This paper followed a two-step research process. First, the collection modes of reusable takeaway containers were designed. Second, the consumers’ and merchants’ preferences for collection modes were analyzed, and the suggestions on the promotion of sustainable consumption was proposed. A flow chart of this two-step research process is shown in Figure 2.

The design of the collection modes of reusable takeaway containers was studied and modified over several rounds. A preliminary design was first developed based on expert interviews and international experiences. Then, the questionnaire surveys were conducted to assess preferences. Questionnaires were distributed to takeaway merchants and consumers to determine their activity, the reasons for their selections, concerns and suggestions. The feasibility and reasonability of the preliminary proposal were analyzed. If it was not reasonable, the preliminary design would be modified, and a new round of design was initiated. Then, a new questionnaire was conducted until the proposal was determined to be appropriate.

The second step of this research involved the analysis of the respondents’ preferences for collection modes. Based on the final round questionnaire results, a binary logistic regression model was applied to determine the factors that influence the consumers’ and merchants’ preferences. According to the results, some suggestions on promoting collection modes were proposed.

### 3.2. Expert Interviews

A panel invited four experts, including a professor from Nanjing Forestry University, a professor from the Southeast University, two senior managers from the takeaway platforms of Meituan takeaway and Eleme. These experts each had more than 10 years of respective field experience, which could make the judgments more consistent and reliable.

The expert interviews were completed in September 2018, December 2018, and June 2019 to provide decision support and advice on five research items: the guidelines for the collection mode; the criteria for judging the reasonableness of the collection mode; several feasible collection modes; a list of questionnaire questions for the questionnaire survey; and the specific survey process.

Taking into account the recommendations of the experts and the literature review [17,18,24,29], we identified the following design guidelines for the collection mode. (1) Protect the environment and make rational use of resources. (2) Ensure food health and safety, and the hygienic status of the containers. (3) Improve consumers’ experience. (4) Take into account business efficiency and cost.

The design of the collection mode was mainly based on national and international cases, the literature review and the experience of experts. There is currently a delivery rider immediate retrieval mode in China. This mode is therefore included in the first round of collection modes. Others are mainly based on the Japanese case and the literature review, in which consumers bring their food containers to a collection point, where they are later picked up by the merchants [23,25,26,27,28]. For this collection mode, we distinguished several different ways to retrieve takeaway containers, namely directly from the consumers by the delivery riders, or from a fixed collection point by the delivery riders. There are also modes that are already emerging in certain European cities, where consumers bring the reusable containers back to the merchants themselves [24]. However, this paper focuses on a takeaway meal, and takeaway consumers usually do not go in-store, so this mode is not taken into account.

In the first round, four collection modes were designed as follows.

Mode 1. The takeaway consumer places the food in their own containers, and returns the empty reusable containers to the delivery driver. Then, the delivery driver returns the containers to the merchants for dishwashing.

Mode 2. The consumer simply washes the containers after a meal, and make an appointment on the app. Then, a delivery driver makes an appointment to pick up the containers and return them to the original takeaway merchant for deep dishwashing.

Mode 3. The consumer simply washes the containers and then brings them to a collection bin for simple automatic dishwashing. Then, a delivery driver picks up the containers and brings them to the merchants for dishwashing.

Mode 4. The consumer brings the food containers to a collection bin for simple automatic dishwashing, and a delivery driver brings them to a dishwashing center for deep dishwashing. The containers are washed and sanitized before being distributed to the merchants.

The experts suggested the probability of the consumers’ and merchants’ choice of collection mode as a criterion for judging the reasonableness of the collection mode. If the selection probability of a collection mode was small relative to several other modes, then this mode can be largely ruled out.

Mode 2, Mode 3 and Mode 4 from the first round were retained, and a new mode was added. The second round of the collection modes were as follows.

Mode A–M. The retained previous Mode 2. Here, the A in Mode A–M is the initial capitalization of the word app and the M is the initial capitalization of the word merchant.

Mode A–W. A new added mode. The consumer simply washes the containers after a meal, and makes an appointment on the app. Then, a driver makes an appointment to pick up the containers and bring them to a dishwashing center for deep dishwashing. Here, the W in Mode A–W is the initial capitalization of the word wash, which means dishwashing center.

Mode B–M. The retained previous Mode 3. The B in Mode B–M is the initial capitalization of the word bin, which means collection bin.

Mode B–W. The retained previous Mode 4.

### 3.3. Preference Questionnaire Design

This paper uses the well established field questionnaire method. This method is simple and effective and has been adopted by the majority of scholars [33,34,35]. In order to investigate the consumers’ and merchants’ preferences, face-to-face questionnaire surveys were conducted with the consumers and merchants, respectively.

The questionnaires for the consumers were collected from takeaway consumers, with answers about their essential information, their ordering takeaway behavior, and their opinions on the preference, concerns and suggestions of promoting reusable takeaway containers [29]. The essential information of the consumers include gender, age, education level and their average monthly income, which are demographic and socioeconomic factors [46]. Their ordering takeaway behavior was surveyed in the perspective of the number of takeaway orders per week, type of takeaway, major reasons for ordering takeaway, average amount spent per meal, delivery requirements for takeaway and the current disposal of takeaway containers [29]. Their opinions on the preference and concerns of promoting reusable takeaway containers were collected, including their willingness to use reusable containers, collection mode choice, reasons for mode preferences and concerns about collection modes. Specific questions were designed to collect the consumers’ suggestions on promoting reusable takeaway containers, namely the acceptable increased costs of reusable takeaway containers, the distance of collection bins, and their willingness to actively participate in a reward system.

The questionnaires for merchants were collected from takeaway merchants, with answers about their essential information, their business characteristics of takeaway and their opinions on the preference, concerns and suggestions of promoting reusable takeaway containers. The essential information of the merchants included gender, age and education level. Their business characteristics of takeaway were surveyed in perspective of catering classification, major forms of catering, daily business hours, number of in-store dinners at peak time, online orders at peak time, the existing distribution mode, number of delivery drivers, and the selection standard of existing containers. Their opinions on the preference, concerns and suggestions of promoting reusable takeaway containers were collected in the same way as that of the consumers’ questionnaire.

Previous studies have used questionnaires, field surveys, and interview surveys. Some have preferred to use mailing questionnaires to identify target respondents, but due to the low return rate (usually less than 40%), the results are always poor [47,48,49]. Some researchers choose to do their surveys online, which is more effective. However, without a face-to-face description, the respondents’ feedback may be distorted [50]. Therefore, we conducted an offline questionnaire and provided enough information to avoid misunderstandings of the respondents. In the process of investigation, we assumed that the respondents were rational people, and would make the best choice for themselves according to their preferences and attitudes [51].

In order to fully understand the consumers’ and merchants’ preferences in reusable takeaway containers, the target respondents are selected among restaurants with high takeaway sales, whose opinions are based on real experience. This questionnaire survey was conducted in four cities, i.e., Nanjing, Jiangyin, Yixing and Yulin. When designing the first-round mode, the responses were collected from 137 consumers and 112 merchants during the second half of 2018. The second-round questionnaire was carried out in the same cities during the first half of 2019. The content of the questionnaire was essentially the same as the first round of the questionnaires, except that the options for several collection modes were modified. A total of 393 consumer questionnaires and 112 merchant questionnaires were collected.

### 3.4. Binary Logistic Regression Model

This paper explores the factors that influence the consumers’ and merchants’ preferences for collection modes by a binary logistic regression model. When the dependent variables are categorical, the linear regression method is not suitable for analyzing the probability of selecting each mode. Therefore, we used a logistic regression model. The dependent variable studied in this paper was “whether to choose a certain mode”, which was divided into “yes” and “no” (coded as “1” and “0”, respectively). Assuming that the random variables follow the logical probability distribution, a binary logistic regression model with *k* independent variables was constructed as follows [44]:

(1) $$P_{i}=\frac{e^{\beta_{0}+\beta_{1}x_{1}+\beta_{2}x_{2}+\ldots \ldots +\beta_{k}x_{k}}}{1+e^{\beta_{0}+\beta_{1}x_{1}+\beta_{2}x_{2}+\ldots \ldots +\beta_{k}x_{k}}}$$

where *P_i_* represents the probability of choosing this mode. *β*_0_, *β*_1_*…β_k_* represent the regression coefficients. *x*_1_, *x*_2_*…x_k_* represent the variables. Because the dependent variable is binary, the error term of the logistic regression model should obey a binomial distribution, and the maximum likelihood method is used to estimate and test the model. It is noteworthy that the significance of each model parameter is less than 0.05, and the parameter modification is effective.

SPSS can be used to filter the independent variables according to forward or backward and stepwise regression. There are three criteria for eliminating independent variables, namely conditional parameter estimation, maximum likelihood estimation, and Wald χ^2^ [45].

## 4. Results

### 4.1. Collection Modes for Reusable Takeaway Containers

#### 4.1.1. First Round of Collection Modes

A first-round questionnaire survey was conducted. A total of 91% of consumers were aware of the impacts of the disposal of takeaway waste. A total of 77% of consumers supported the use of reusable food containers, but they were concerned about dishwashing and disinfection. A total of 70% of consumers used takeaway services because it is convenient and saves time. However, returning the containers to the collection bins requires additional time; a total of 57% of consumers were not willing to spend time on this. Consumers were more inclined to support collection modes that take less time. According to the results of the first round of the performance survey, which are shown in Table 1, Mode 2, Mode 3 and Mode 4 have great advantages in terms of consumer experience and hygiene, and thus, these modes could be retained. However, Mode 1 involve poor consumer experience and would be difficult to promote. Therefore, a second round of mode design was required.

#### 4.1.2. Second Round of Collection Modes

**Figure 3:**
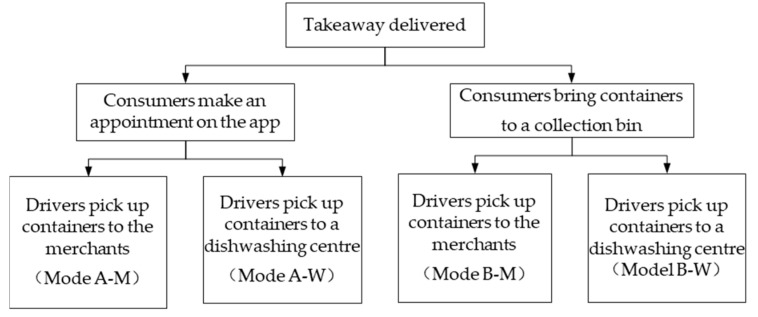
Determined collection modes.

A second-round questionnaire survey was carried out. The preferences for the second round of the modes are shown in Table 2. As shown in Table 2, the merchants and consumers had similar preferences for the four modes. Both merchants and consumers tended to prefer using dishwashing centers to ensure hygiene. Moreover, all four modes have a certain applicability and an intended population, so the four modes shown in Figure 3 were finally determined as promotion modes.

For the consumers, there were two preferences, namely make an appointment on the app (Mode A–M and Mode A–W, called Mode A for short) or bring the containers to a collection bin for dishwashing (Mode B–M and Mode B–W, called Mode B for short). For the merchants, there were two preferences, namely return the containers to the merchants (Mode A–M and Mode B–M, called Mode M for short) or bring the containers to a dishwashing center (Mode A–W and Mode B–W, called Mode W for short).

### 4.2. Regression Model Results

#### 4.2.1. Regression Analysis Results of Consumers’ Preference

The SPSS 25.0 was used to filter the independent variables according to forward or backward and stepwise regression. Thirteen variables were defined, and the statistical results of the consumers’ survey sample are shown in Table 3. A binary logistic model was applied by taking the collection modes as the dependent variable, namely Mode A and Mode B. The regression coefficient and significance of thirteen variables are shown in Table 3.

The likelihood ratio test and the Hosmer–Lemeshow test were used for the goodness-of-fit analysis, and the results are shown in Table 4. The larger the R^2^ value is, the better the fit of the model is [45]. The values of Cox–Snell R^2^ and Nagelkerke’s R^2^ were larger than 0.05, indicating that the model fits well [44]. The Hosmer–Lemeshow test outputs are the chi-square statistic, df and significance [45]. In this model, the significance was 0.454, which is greater than the significance level of 0.05. Therefore, the difference between the comparison groups of the consumers’ regression model was not significant. That is, the independent variables defined are effective, and the model construction is meaningful.

#### 4.2.2. Regression Analysis Results of Merchants’ Preferences

The SPSS 25.0 was used to filter the independent variables according to forward or backward and stepwise regression. Fourteen independent variables were defined, and the statistical results of the merchant survey sample are shown in Table 5. Binary logistic regression was applied by taking the collection modes as the dependent variable, namely Mode M and Mode W. The regression coefficient and significance of each variable are shown in Table 5.

The likelihood ratio test and the Hosmer–Lemeshow test were used for the goodness-of-fit analysis, and the results are shown in Table 6. The significance was 0.01, which indicates that the original hypothesis was rejected. That is, the difference between the comparison groups of the merchant model was significant. The significance of the chi-square statistic was less than 0.05, indicating a poor fit. Because 70% of the merchants tend to choose Mode W, the individual characteristics have little impact on the preferences.

### 4.3. Questionnaie Results of Consumers’ and Merchants’ Concerns and Suggestions

As can be seen from Table 3 and Table 5, the consumers of takeaway are more concerned about the hygienic status of the takeaway containers, the distance of the collection bins, the increased cost, and the merchants of takeaway are more concerned about the hygienic status of the takeaway containers and the increased cost.

The consumers’ suggestions on promoting reusable takeaway containers were collected from the questionnaire, and focused on the distance of dishwashing facilities and acceptable increased costs.

**Figure 4:**
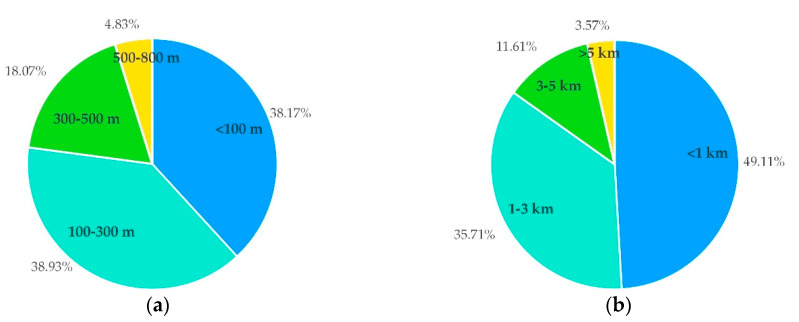
The preferences for the distance of dishwashing facilities, (**a**) for the consumers and (**b**) for the merchants.

The consumers’ preferences for the distance of collection bins is shown in Figure 4a. A total of 38% of the consumers preferred a distance under 100 m. Figure 4b shows the merchants’ preferences for the distance of dishwashing centers. A total of 49% of the merchants preferred a distance under 1 km, which can reduce the delivery delay and not affect the normal operation of the restaurant. However, 36% of the merchants believed that the distance between the dishwashing center and the restaurant should be 1–3 km. This preference for more distance was due to factors such as the discharge of sewage from dishwashing centers and the irritating smell of disinfecting water.

**Figure 5:**
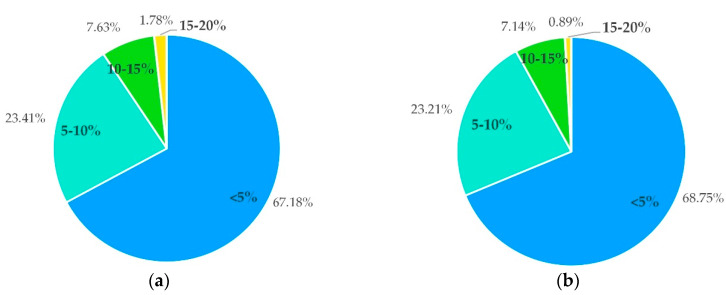
The preferences for an increase in costs, (**a**) for the consumers and (**b**) for the merchants.

Compared to the disposable takeaway containers, the purchase and collection of reused containers and the operation of dishwashing facilities are all direct contributors to the increased costs of takeaway businesses. Indirectly, they contribute to the increased cost of ordering takeout for consumers. Both the consumers and merchants are concerned about the increased cost. As shown in Figure 5, the consumers and merchants basically have the same opinions on an acceptable increased cost. The most acceptable increased cost is less than 5%, as reported by 67% and 69% of consumers and merchants, respectively. The merchants were willing to participate in this collection program if a balance was found between the benefits of environmental protection by using reusable takeaway containers and minimizing costs as much as possible. The questionnaire results show that 54% of merchants and 75% of consumers would be willing to actively participate in a reward system.

## 5. Discussion

### 5.1. Discussion of Factors Influencing Consumers’ and Merchants’ Preference

In this study, we found the impacts of the independent variables on the consumers’ and merchants’ preferences. The independent variables of the consumers’ preferences, shown in red in Table 3, were the significance influencing factors with significance values of less than 0.05, and included “gender”, “delivery requirements”, “current disposal of takeaway containers”, “reasons for mode preferences”, and “concerns about modes”, which indicates that these variables had stronger effects on the dependent variables. Among them, the variables “delivery requirements” and “current disposal of takeaway containers” had a significant impact on the consumers’ preferences. The relationship between the different attributes of independent variables and the consumers’ preferences of collection modes are discussed as follows, and summarized in Table 7.

Consumers under the age of 20 were more likely to select Mode B. Most of them were university students or young workers who did not find it convenient to wash dishes, and therefore, they preferred to send the containers to a small dishwashing bin. Consumers aged between 21 and 50 preferred Mode A, as it was easier for these individuals to wash their dishes.

Consumers who ordered takeaway 1–2 times and 3–4 times a week preferred Mode A. The average amount spent per meal was mostly in the CNY 15 and CNY 15–30 categories. Consumers spending less than CNY 15 per meal were more likely to select Mode B, but those spending between CNY 30–50 per meal were less likely to select Mode B.

Consumers who chose Mode B were more concerned about hygiene and the distance of small automatic dishwashing centers.

The impacts of the independent variables on the merchants’ preferences were shown in Table 5. The significant influencing factors are the independent variables with significance values less than 0.05, which are shown in red in Table 5, namely “catering classification”, “major forms of catering”, “concerns about collection mode” and “distance of a dishwashing center”, indicating that these variables had stronger effects on the dependent variables. The relationship between the different attributes of the independent variables and the merchants’ preferences are shown in Table 8, and are described as follows.

Merchants offering burgers, pizza and dessert drinks preferred Mode M. Hamburgers and pizzas are packaged in simple disposable paper bags, which essentially eliminates the need for reusable containers. Therefore, these merchants preferred Mode M due to its lower cost. Our goal, of course, is to promote the use of reusable containers or the packaging of all types of food, thus preventing packaging waste, even if the packaging is easily discarded. Foods such as pizza and hamburgers can also use reusable containers or packages.

The longer the business hours were, the more likely it was that merchants would choose Mode W. The more consumers there were in the restaurant at peak dining hours, the more likely it was that the merchants would choose Mode M, probably to avoid running out of containers in the restaurant. The more consumers there were ordering online at peak time, the more likely it was that the merchants would choose Model W. When there were high numbers of both online ordering consumers and in-store dining consumers, the merchants were more likely to choose Mode W. A dishwashing center ensures both hygiene and the timely turnover of containers.

According to the above discussion, consumers and merchants of different ages, genders and consumption levels have different preferences for the modes. Several factors influence the consumers’ and merchants’ choices. Therefore, different collection modes should be promoted for different crowds. Considering the high cost of setting up collection bins, it is recommended that these bins be set up where the takeaway consumers are concentrated.

For consumers, collection modes can be promoted depending on the number of takeout times, the average amount spent per meal and age. Consumers who order takeaway more often and spend more preferred Mode B. For consumers who are young students, Mode B should be promoted in dormitories on campuses. If it is convenient for consumers to wash their dishes, such as in residential areas, Mode A should be promoted.

For takeaway merchants, collection modes can be promoted depending on the categories of catering and the number of consumers. Mode M is recommended for merchants who sell food with simple packaging, such as hamburgers, pizza and dessert drinks. Mode M is also recommended for merchants with low-peak dining attendance; conversely, Mode W is recommended for those with high-peak dining attendance.

### 5.2. Suggestions on Promoting Collection Modes

To promote the collection mode of reusable takeaway containers, some issues need to be addressed, namely, the locations of dishwashing facilities, the increased costs and the hygienic status of takeaway containers.

It is recommended that the collection bin be within 500 m of the consumers and the dishwashing center be within 5 km of the merchant.

In response to the increased operation costs, a reward system is recommended based on the experience in Japan [22]. Takeaway platforms and other organizations could reward merchants and consumers with points for completing the collection of reusable takeaway containers once. 

In addition, it is recommended to seek some support and help from the government administrative departments. The promotion of relevant policies requires all parties to effectively participate in the use of reusable containers to ensure the smooth implementation of the collection process. The tax bureau can provide a certain degree of benefits to merchants in terms of tax revenue. The environmental protection bureau can implement a carbon credit system for merchants to offset part of the tax so that the operation costs of merchants can be reduced.

To implement the collection modes of reusable takeaway containers, the government regulatory departments and takeaway platforms need to formulate a series of articles of association and publicity. Thus, the merchants and consumers would have no doubts about the hygiene and costs and would actively participate in the use and promotion of reusable takeaway containers.

It is suggested that the health supervision department be transparent in establishing strict regulations and evaluation mechanisms to standardize the production and disinfection process of containers. The food safety office needs to strengthen the supervision and spotcheck process, cooperating with the administration of the industry and commerce to punish manufacturers who illegally produce low-quality containers and those who neglect the dishwashing and disinfection process. It is important that consumers trust the reusable containers and their sanitation and that they are easily able to use the containers.

## 6. Conclusions

This paper presented designs for collection modes for reusable takeaway containers, and analyzed the takeaway consumers’ and merchants’ preferences for collection modes. Based on the preferences and concerns of consumers and merchants, we proposed promotion strategies and guarantee measures, such as different promotion strategies for different people, suggestions on the locations of dishwashing facilities, reward systems and food safeguard measures.

It is recommended that different collection modes should be promoted for different people. For takeaway merchants, collection modes can be promoted depending on the categories of catering and the number of consumers. For the consumers, collection modes can be promoted depending on the number of takeout times and the average amount spent per meal and age. It is also recommended that the collection bin be within 500 m of the consumers and the dishwashing center be within 5 km of the merchant. In response to the increased operation costs, a reward system is recommended. The environmental protection bureau can implement a carbon credit system for the merchants to offset part of the tax so that the operation costs of merchants can be reduced. It is suggested that the health supervision department be transparent in establishing strict regulations and evaluation mechanisms to standardize the production and disinfection process of reusable food containers.

However, there are still some limitations. The number of merchants surveyed herein was too small, which led to deviations in the fit of the model. In the future, we will continue to identify the influencing factors and conduct in-depth research to develop models with better fit. In addition, since takeaway consumers do not typically go to in-store dinner, this study did not consider the possibility of consumers bringing the reusable food containers back to the merchants. However, when promoting the collection of all food containers in the future, the feasibility of this mode will need to be considered.

## Figures and Tables

**Table 1:** The preferences of the first-round modes.

Object	Mode 1	Mode 2	Mode 3	Mode 4
Merchants	0%	21.43%	44.64%	33.93%
Consumers	1.64%	40.98%	31.15%	26.23%

**Table 2:** The preferences of the second-round modes.

Object	Mode A–M	Mode A–W	Mode B–M	Mode B–W
Merchants	22.32%	25.89%	9.83%	41.96%
Consumers	21.37%	26.46%	18.33%	33.84%

**Table 3:** Characteristics of the consumer survey samples and the regression analysis results of the consumers’ preferences.

Variable	Characteristics (Assignment)	Proportion (%)	Coefficient	Significance
Gender (V1)	Male (1)	35.37	0.004	**0.045**
	Female (2)	64.63		
Age (V2)	<20 years old (1)	24.43	0.502	0.063
	21–35 years old (2)	47.84	−0.333	0.103
	35–50 years old (3)	25.45	−0.103	0.97
	>50 years old (4)	2.29		
Education level (V3)	Primary education (1)	1.02	21.993	0.054
	Junior high school (2)	5.85	−0.381	0.197
	High school (3)	13.49	−0.765	0.095
	University degree (4)	72.77	0.054	0.275
	Graduate degree (5)	6.87		
Average monthly income (V4)	<CNY 1000 (1)	31.3	−0.448	0.801
	CNY 1000–CNY 2000 (2)	19.08	−0.321	0.975
	CNY 2000–CNY 4000 (3)	18.07	0.035	0.786
	CNY 4000–CNY 6000 (4)	14.25	−0.07	0.726
	>CNY 6000 (5)	17.3		
Number of takeaway orders per week (V5)	0 time (1)	18.58	0.297	0.201
	1–2 times (2)	46.56	−0.487	0.269
	3–4 times (3)	19.34	−0.27	0.674
	>5 times (4)	15.52		
Average amount spent per meal (V6)	<CNY 15 (1)	29.26	0.144	0.373
	CNY 15–CNY 30 (2)	50.38	0.057	0.796
	CNY 30–CNY 50 (3)	16.28	−0.427	0.514
	>CNY 50 (4)	4.07		
Delivery requirement (V7)	Delivered to consumers (1)	64.89	0.574	**0.042**
	Place in designated place (2)	35.11		
Current disposal (V8)	Classified disposal (1)	26.97	−0.626	**0.033**
	Disposal with other garbage (2)	73.03		
Willingness to use reusable containers (V9)	Yes (1)	75.06	0.04	0.794
	No (2)	7.12	−0.823	0.071
	It depends (3)	17.81		
Reasons for mode preferences (V10)	Convenient and efficient (1)	27.48	−0.759	0.212
	Strong enforceability (2)	36.64	0.244	0.389
	Good hygienic status (3)	30.53	1.756	**0.003**
	Others (4)	5.34		
Concerns about collection mode (V11)	Hygienic status (1)	49.36	0.299	0.397
	Increased costs (2)	13.99	0.409	0.283
	Distance of collection bins (3)	16.03	1.474	**0.025**
	Insufficient containers (4)	8.4	0.335	0.163
	Broken containers (5)	6.62	0.739	0.446
	Others (6)	5.6		
Acceptable increased costs (V12)	<5% (1)	67.18	0.514	0.117
	5–10% (2)	23.41	0.095	0.153
	10–15% (3)	7.63	−0.171	0.805
	15–20% (4)	1.78		
Distance of collection bins (V13)	<100 m (1)	39.45	−0.966	0.087
	100–300 m (2)	37.37	−0.302	0.385
	300–500 m (3)	17.99	−0.461	0.8
	500–800 m (4)	5.19		
Constant			−0.219	0.087

**Table 4:** Goodness-of-fit results of the consumers’ regression model.

−2 Log Likelihood	Cox–Snell R^2^	Nagelkerke’s R^2^	Chi-Square	Df	Significance
431.474	0.249	0.332	7.791	8	0.454

**Table 5:** Characteristics of the merchant survey samples and the regression analysis results of the merchants’ preferences.

Variable	Characteristics (Assignment)	Proportion (%)	Coefficient	Significance
Gender (T1)	Male (1)	45.54	0.32	0.571
	Female (2)	54.46		
Age (T2)	<25 years old (1)	33.04	0.002	0.963
	25–40 years old (2)	49.11	2.216	0.137
	41–60 years old (3)	15.18	−0.43	0.153
	>60 years old (4)	2.68		
Education level (T3)	Primary education (1)	8.93	1.605	0.205
	Junior high school (2)	8.04	0.442	0.506
	High school (3)	22.32	0	0.986
	University degree (4)	57.14	0.413	0.521
	Graduate degree (5)	3.57		
Catering classification (T4)	Hotel (1)	10.71	4.227	**0.04**
	Fast food restaurant (2)	41.07	5.662	**0.017**
	Snack bar (3)	35.71	0.615	0.433
	Beverage shop (4)	12.5		
Major forms of catering (T5)	Simple meals (1)	44.64	0.458	0.399
	Noodle and gruel (2)	31.25	0.965	0.326
	Western fast food (3)	22.32	−0.906	0.639
	Japanese, Korean cuisine (4)	14.29	1.823	0.215
	Fried snacks (5)	17.86	0.921	0.45
	Fruits and fresh food (6)	10.71	0.477	0.926
	Sweets and drinks (7)	21.43	−2.075	**0.009**
Daily business hours (T6)	<12 h (1)	52.68	0.681	0.409
	12–16 h (2)	40.18	1.034	0.309
	>16 h (3)	7.14		
Number of in-store diners at peak time (one hour) (T7)	<20 person time (1)	41.96	0.206	0.65
	20–50 person time (2)	33.93	0.269	0.604
	50–100 person time (3)	17.86	−0.116	0.406
	>100 person time (4)	6.25		
Online orders at peak (one hour) (T8)	<20 orders (1)	44.64	0.001	0.977
	50–100 orders (2)	44.64	0.19	0.663
	>100 orders (3)	10.71		
Existing distribution mode (T9)	Merchants’ distribution (1)	21.43	1.791	0.181
	Platform distribution (2)	50.89	1.175	0.278
	Both (3)	27.68		
Number of delivery drivers (T10)	<3 person (1)	52.68	0	0.988
	3–8 person (2)	38.39	0.006	0.941
	>8 person (3)	8.93		
Selection standard of existing containers (T11)	Price (1)	25.89	0.601	0.438
	Quality (2)	38.39	4.023	0.045
	Hygiene (3)	23.21	3.048	0.081
	Environmental (4)	12.5		
Reasons for mode preferences (T12)	Convenient and swift (1)	37.5	0.044	0.834
	Strong implementation (2)	25	0	1
	Good hygienic status (3)	27.68	0.19	0.663
	Others (4)	9.82		
Concerns about collection mode (T13)	Hygienic status (1)	34.82	7.537	**0.006**
	Increased costs (2)	25	0.218	0.64
	Distance of dishwashing center (3)	5.36	0.696	0.404
	Insufficient containers (4)	13.39		
Distance of dishwashing center (T14)	<1 km (1)	49.11	6.545	**0.011**
	1–3 km (2)	35.71	1.455	0.228
	3–5 km (3)	11.61		
Constant			0.596	0.828

**Table 6:** Goodness-of-fit results of the merchants’ regression model.

−2 Log Likelihood	Cox–Snell R^2^	Nagelkerke’s R^2^	Chi-Square	Significance
106.307	0.264	0.369	20.096	0.010

**Table 7:** Relationship between the attributes of independent variables and the consumers’ preferences.

Attributes of Independent Variables	Consumers’ Preference of Collection Mode	
	Mode A	Mode B
Delivery requirement	/	Delivered to consumers
Current disposal	Classified disposal	/
Age	21–50 years old	<20 years old
Number of takeout times per week	1–2 times	/
Average amount spent per meal	<CNY 30	CNY 30–CNY 50

**Table 8:** Relationship between the attributes of the independent variables and the merchants’ preferences.

Attributes of Independent Variables	Merchants’ Preference of Collection Mode	
	Mode M	Mode W
Catering classification	/	Fast food restaurant, hotel
Major forms of catering	Burgers, pizza and dessert drinks	/
Daily business hours	/	Longer
In-store diners at peak time	More diners	/
Online orders at peak	/	More orders
